# Survival from cancer of the uterine cervix in England and Wales up to 2001

**DOI:** 10.1038/sj.bjc.6604589

**Published:** 2008-09-23

**Authors:** M J Quinn, N Cooper, B Rachet, E Mitry, L M Woods, M P Coleman

**Affiliations:** 1 Social and Health Analysis and Reporting Division, Office for National Statistics (Room FG/114), 1 Myddelton Street, London EC1R 1UW, UK; 2 Cancer Research UK Cancer Survival Group, Non-Communicable Disease Epidemiology Unit, Department of Epidemiology and Population Health, London School of Hygiene and Tropical Medicine, Keppel Street, London WC1E 7HT, UK; 3 Département d'Hépatogastroentérologie et Oncologie Digestive, Centre Hospitalo-Universitaire Ambroise-Paré, 9 avenue Charles de Gaulle, F-92100 Boulogne, France

Cervical cancer is the third most common cancer in women worldwide, after cancers of the breast and large bowel, with approximately 470 000 new cases a year in 2000, some 10% of all cancers in women (Ferlay *et al*, 2001). Approximately 80% of all cases occur in developing countries, where cervical cancer is often the most common cancer in women, whereas in developed countries, cervical cancer only accounts for about 5% of all cancers in women. It has been known for 30 years from observational data that cervical cancer is likely to have an infectious component (Beral, 1974), subsequently confirmed as HPV (Bosch *et al*, 1994). Trials of vaccination already appear promising for primary prevention (Gravitt and Shah, 2005; Mao *et al*, 2006).

In England and Wales, the age-standardised incidence of cervical cancer in the 1970s and 1980s remained between 14 and 16 per 100 000, although age-specific rates were influenced by strong cohort effects (those women born in the 1890s were at high risk, those born in the mid-1930s at low risk). The cervical screening programme began in the 1960s, offering cervical smear tests to detect premalignant lesions in women aged 25–64 years on a 5-yearly basis, but it was largely ineffective until the late 1980s (Murphy *et al*, 1988). In 1988, a national call and re-call system was established and coverage rose rapidly to approximately 85% (Quinn *et al*, 1999). As a result of these improvements, the incidence of invasive malignancy fell continuously after 1990 to below 9 per 100 000 in 2000 (Quinn *et al*, 2001; Office for National Statistics, 2003a).

Mortality from cervical cancer in England and Wales had been declining steadily at approximately 1.5% each year from the early 1950s to the late 1980s. This long-term decline began before the introduction of screening. From 1990, the rate of decline in mortality increased three-fold, and by 2002 the age-standardised death rate had fallen to 29 per 100 000, barely one-third of the death rate in 1971 (83 per 100 000) (Office for National Statistics, 1999, 2003b). Only 1000 deaths were attributed to cervical cancer in 2002, and the screening programme has been credited with preventing 800 or more deaths a year (Quinn *et al*, 1999; Peto *et al*, 2004).

We analysed the data for 44 090 women diagnosed with invasive cancer of the uterine cervix in England and Wales during the 14-year period 1986–1999, some 90% of those eligible for inclusion in the analyses. Approximately 4% of women otherwise eligible for analysis were excluded with zero recorded survival (date of diagnosis same as date of death). The proportion of women for whom recorded survival was zero did not vary between socioeconomic groups, however, and it was stable throughout the 1990s (data not shown). Therefore, these exclusions are unlikely to have had any substantial impact on socioeconomic gradients in survival, or on trends in the gradient. A further 3% of women with cervical cancer were excluded because it was not their first primary malignancy, and 2% because their vital status was unknown on 5 November 2002, the date when the data were extracted for analysis.

Some two-thirds (67%) of cervical cancers were squamous carcinomas, and a further 16% were adenocarcinomas: both proportions were similar throughout the 1990s. Other morphological types were individually rare, but cancers with poorly specified morphology accounted for about 10%.

Annual incidence rates for invasive cervical cancer in the late 1990s were 50% higher among the most deprived women (12.8 per 100 000) than among the most affluent (8.5 per 100 000), but this difference was less marked than in the early 1990s (63%). This is because incidence declined more among the most deprived women over this period (18%) than among the most affluent (11%) (Figure 1).

## Survival trends

Survival from cervical cancer is moderate: in the early 1970s, 1-year relative survival was 75% and 5-year survival just over 50% (Coleman *et al*, 1999). During the 1970s and 1980s, 1-year survival improved by more than 2% every 5 years, reaching 83% for women diagnosed during 1986–1990. Five-year survival improved more rapidly, by more than 3% every 5 years, to reach 64% by the same period.

**Figure 2:**
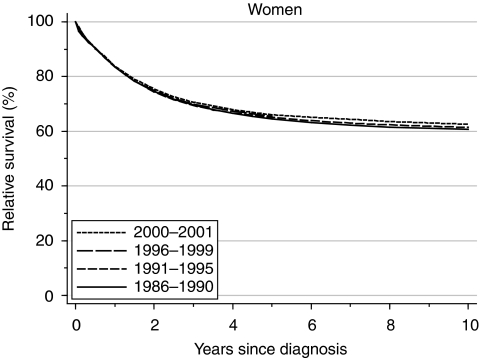
Relative survival (%) up to 10 years after diagnosis by calendar period of diagnosis: England and Wales, adults (15–99 years) diagnosed during 1986–1999 and followed up to 2001. Survival estimated with cohort or complete approach (1986–1990, 1991–1995, 1996–1999) or hybrid approach (2000–2001) (See Rachet *et al*, 2008).

In the 1990s, by contrast, 1-year and 5-year survival did not increase significantly, remaining at 85.5 and 65.5%, respectively (Table 1, Figure 2). The small average increase in 1-year survival (0.5% every 5 years) is adjusted for the deprivation gap in survival and for changes in the distribution of patients by deprivation category (see above). It is a more reliable estimate of trend than would appear from the unchanging estimates of 83.5% in successive calendar periods, although the rate of increase in survival is not statistically significant.

Short-term predictions of survival up to 10 years after diagnosis, based on hybrid analysis of patients' survival experience during 2000–2001 (Brenner and Rachet, 2004) suggest that improvement in survival in the near future is unlikely (Table 1); this reflects the lack of recent improvements in early survival.

## Deprivation

During the 1970s and 1980s, relative survival from cervical cancer in England and Wales was consistently some 4–8% lower for women living in deprived areas than for those in more affluent areas (Coleman *et al*, 1999). This deprivation gap did not appear to be accounted for by differences in stage at diagnosis (Schrijvers *et al*, 1995).

**Figure 3:**
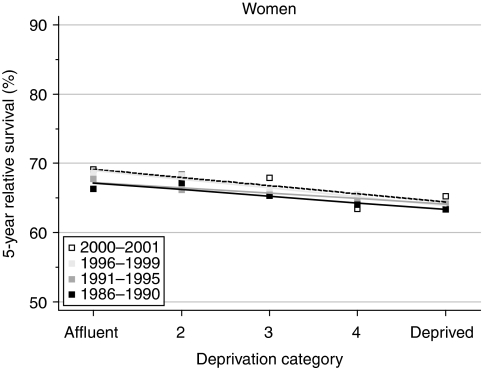
Trends in the deprivation gap in 5-year relative survival (%) by calendar period of diagnosis: England and Wales, adults (15–99 years) diagnosed during 1986–1999 and followed up to 2001.

For women diagnosed in the late 1980s, the deprivation gap in survival was just under 3% at 1 year after diagnosis and 4% at 5 years. The deprivation gap in 5-year survival became slightly but significantly wider during the 1990s, reaching 4–5% for women diagnosed during 1996–1999 (Table 2, Figure 3).

Short-term predictions of survival by socioeconomic group, using hybrid analysis of the probabilities of survival observed during 2000–2001, do not suggest any imminent change in the deprivation gradient in survival (Table 2).

## Comment

Following improvements to the cervical screening programme in the late 1980s, the incidence of invasive cervical cancer and the death rate both fell sharply – but survival from cervical cancer has not improved further during the 1990s, and there is no evidence that any increase can be expected in the near future. The differences in survival between affluent and deprived women have been persistent for many years.

The lack of progress in the 1990s seems difficult to explain with the available data. If the steady improvements in survival seen in the 1970s and 1980s did in fact continue for women diagnosed at each stage of disease during the 1990s, then the absence of any observed trend in overall survival (all stages of disease combined) may have been attributable to changes in the distribution of age or stage at diagnosis.

Carcinoma *in situ* of the cervix was detected increasingly often during the 1990s in women aged 20–34 years, but not in older women (Quinn *et al*, 2001). In most women, effective treatment of carcinoma *in situ* would have prevented progression of the disease to invasive cancer: in that sense, cervical screening can actually prevent the development of invasive cancer that would otherwise have arisen. Women with carcinoma *in situ*, predominantly young women, were not included in these analyses, and the overall survival trends for women aged 15–99 years, who *were* ultimately diagnosed with invasive cancer, did not change significantly by standardisation for age (data not shown here). Therefore, a shift towards an older age distribution of women diagnosed with invasive cervical cancer cannot explain the lack of improvement in overall survival.

The spectrum of stage at diagnosis of invasive cancer may have shifted towards more advanced cases, as a result of continuing improvement in the screening programme. As a result, any improvement in survival at each stage of disease may have been somewhat masked in the overall survival figures for all women with invasive cancer of the cervix. The need for high-quality data on stage of diagnosis to enable adequate evaluation of national trends in cancer survival is obvious, but those data can only be captured by cancer registries if the relevant information is systematically recorded in the medical record for all cancer patients.

The decline in the incidence and mortality of invasive cervical cancer is a very welcome result of improved cervical screening. Primary prevention by vaccination against HPV also appears to have great promise within the next decade. Despite this evidence of solid progress in the control of cervical cancer, 1000 deaths a year is still far too many for a disease that has been largely preventable for decades. It remains a cause for concern that no significant improvement in survival from cervical cancer has occurred since the late 1980s, and that socioeconomic inequalities in survival have tended to worsen rather than improve.

## Figures and Tables

**Figure 1:**
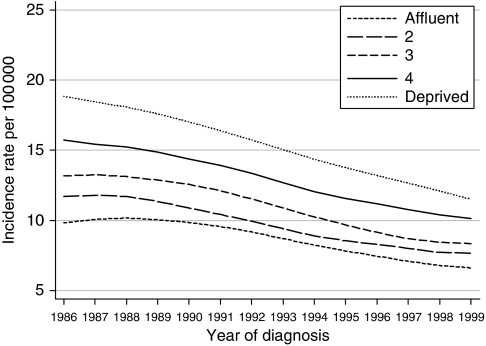
Trends in the age-standardised incidence of cervical cancer in women aged 15–99 years, by deprivation group: England and Wales, 1986–1999.

**Table 1:** Trends in relative survival (%) by time since diagnosis and calendar period of diagnosis: England and Wales, adults (15–99 years) diagnosed during 1986–1999 and followed up to 2001

		**Calendar period of diagnosis^a^**									
		**1986–1990**		**1991–1995**		**1996–1999**		**Average change (%) every 5 years^b^**		**Prediction^c^ for patients diagnosed during 2000–2001**	
**Time since diagnosis**		**Survival (%)**	**95% CI**	**Survival (%)**	**95% CI**	**Survival (%)**	**95% CI**	**Survival (%)**	**95% CI**	**Survival (%)**	**95% CI**
1 year	Women	**83.5**	(82.9, 84.0)	**83.5**	(82.9, 84.1)	**83.5**	(82.7, 84.2)	**0.5**	(−0.9, 1.8)	**83.8**	(82.7, 84.8)
5 years	Women	**64.5**	(63.8, 65.3)	**65.1**	(64.2, 65.9)	**65.5**	(64.4, 66.6)	**0.9**	(−1.1, 2.8)	**66.0**	(64.5, 67.4)
10 years	Women	**60.7**	(59.9, 61.5)	**61.4**	(60.4, 62.3)			**−1.7**	(−5.2, 1.7)	**62.6**	(61.0, 64.0)

CI=confidence interval.

a Survival estimated with cohort or complete approach (see Rachet *et al*, 2008).

b Mean absolute change (%) in survival every 5 years, adjusted for deprivation (see Rachet *et al*, 2008).

c Survival estimated with hybrid approach (see Rachet *et al*, 2008).

**Table 2:** Trends in the deprivation gap in relative survival (%) by time since diagnosis and calendar period of diagnosis: England and Wales, adults (15–99 years) diagnosed during 1986–1999 and followed up to 2001

		**Calendar period of diagnosis^a^**									
		**1986–1990**		**1991–1995**		**1996–1999**		**Average change (%) every 5 years^b^**		**Prediction^c^ for patients diagnosed during 2000–2001**	
**Time since diagnosis**		**Deprivation gap (%)**	**95% CI**	**Deprivation gap (%)**	**95% CI**	**Deprivation gap (%)**	**95% CI**	**Deprivation gap (%)**	**95% CI**	**Deprivation gap (%)**	**95% CI**
1 year	Women	−2.8**	(−4.5, −1.1)	−3.6**	(−5.5, −1.8)	−3.7**	(−5.9, −1.5)	**−0.5**	(−1.9, 0.9)	−3.6*	(−6.7, −0.4)
5 years	Women	−3.8**	(−6.1, −1.6)	−3.1*	(−5.6, −0.7)	−5.1**	(−8.4, −1.7)	**−0.4**	(−2.4, 1.6)	−4.8*	(−9.0, −0.5)
10 years	Women	−5.3**	(−7.6, −2.9)	**−2.5**	(−5.3, 0.2)			**2.7**	(−0.9, 6.4)	**−3.8**	(−8.2, 0.7)

CI=confidence interval.

a Survival estimated with cohort or complete approach (see Rachet *et al*, 2008).

b Mean absolute change (%) in the deprivation gap in survival every 5 years, adjusted for the underlying trend in survival (see Rachet *et al*, 2008).

c Survival estimated with hybrid approach (see Rachet *et al*, 2008).

^*^P<0.05; ^**^P<0.01.
